# Semilocal Meta-GGA Exchange–Correlation Approximation from Adiabatic Connection Formalism: Extent and Limitations

**DOI:** 10.1021/acs.jpca.3c03976

**Published:** 2023-10-09

**Authors:** Subrata Jana, Szymon Śmiga, Lucian A. Constantin, Prasanjit Samal

**Affiliations:** α Department of Chemistry & Biochemistry, The Ohio State University, Columbus, Ohio 43210, United States; β Institute of Physics, Faculty of Physics, Astronomy and Informatics, Nicolaus Copernicus University in Toruń, ul. Grudzikadzka 5, 87-100 Toruń, Poland; § Istituto di Nanoscienze, Consiglio Nazionale delle Ricerche CNR-NANO, 41125 Modena, Italy; ∥ School of Physical Sciences, National Institute of Science Education and Research, HBNI, Bhubaneswar 752050, India

## Abstract

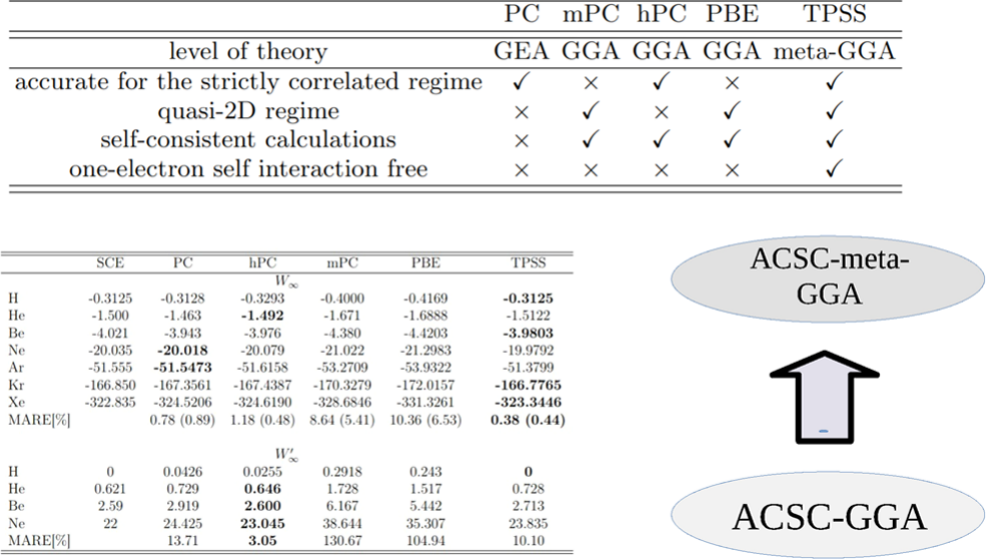

The incorporation of a strong-interaction regime within the approximate semilocal exchange–correlation functionals still remains a very challenging task for density functional theory. One of the promising attempts in this direction is the recently proposed adiabatic connection semilocal correlation (ACSC) approach [ConstantinL. A.; Phys. Rev. B2019, 99, 085117] allowing one to construct the correlation energy functionals by interpolation of the high and low-density limits for the given semilocal approximation. The current study extends the ACSC method to the meta-generalized gradient approximations (meta-GGA) level of theory, providing some new insights in this context. As an example, we construct the correlation energy functional on the basis of the high- and low-density limits of the Tao–Perdew–Staroverov–Scuseria (TPSS) functional. Arose in this way, the TPSS-ACSC functional is one-electron self-interaction free and accurate for the strictly correlated and quasi-two-dimensional regimes. Based on simple examples, we show the advantages and disadvantages of ACSC semilocal functionals and provide some new guidelines for future developments in this context.

## Introduction

The electronic structure calculations of quantum chemistry, solid-state physics, and material sciences become enormously simple since the advent of the Kohn–Sham (KS)^1,2^ density functional theory (DFT).^3^ In DFT, the development of an efficient yet accurate exchange–correlation (XC) functional, which contains all the many-body quantum effects beyond the Hartree method, is one of the main research topics since the last couple of decades and continues to be the same in recent times. The accuracy of the ground-state properties of electronic systems depends on the XC functional approximation (density functional approximation—DFA). The nonempirical XC functionals are developed by satisfying many quantum mechanical exact constraints^4−7^ such as density scaling rules of XC functionals due to coordinate transformations,^5,8−10^ second (and fourth) order gradient expansion of exchange and correlation energies,^11−17^ low density, and high density limit of the correlation energy functional,^18−20^ asymptotic behavior of the XC energy density or potential,^21−28^ quasi-two-dimensional (quasi-2D) behavior of the XC energy,^29−32^ and exact properties of the XC hole.^7,33−35^

Different rungs of Jacob’s ladder^36^ classification of nonempirical XC approximations are developed based on the use of various ingredients, from the simple spin densities and their gradients, until the occupied and unoccupied KS orbitals and energies.^37−43^ The first rung of the ladder is the local density approximations (LDA).^1^ Next rungs are represented by semilocal (SL) functionals, such as generalized gradient approximations (GGA)^44,45^ and meta-GGA.^6,7,46−51^ Higher rungs are known as 3.5 rung XC functionals,^52−58^ hybrids and hyper-GGAs,^59−75^ double hybrids,^76−81^ and adiabatic connection (AC) random-phase approximation (RPA) like methods and DFT version of the coupled-cluster theory.^13,31,39,40,82−91^

Specifically, we recall that the AC formalism,^92−98^ used in various sophisticated XC functionals,^82,89,94−110^ is based on the coupling-constant (or interaction strength) integral formula^92,94−97^
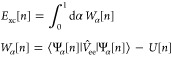
1where *V̂*_ee_ is the Coulomb operator, *U*[*n*] is the Hartree energy, Ψ_α_[*n*] is the antisymmetric wave function that yields the density *n*(**r**) and minimizes the expectation value ⟨*T̂* + α*V̂*_ee_⟩, with *T̂* being the kinetic energy operator, and α is the coupling constant. Equation 1 can be seen as the exact definition of the XC functional, and it connects a noninteracting single particle system (α = 0) to a fully interacting one (α = 1). Note that the α → 0 limit is known as the weak-interaction limit (or high-density or *r*_s_ → 0 limit, where *r*_s_ is the local Seitz radius), where the perturbative approach is valid. Thus, the well-known second-order Görling–Levy perturbation theory (GL2)^18−20,111^ can be applied in the weak-interaction limit, and *W*_α_[*n*] can be expanded as^43^
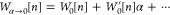
2where *W*_0_ = *E*_x_ and *W*_0_^′^[*n*] = 2*E*_c_^GL2^[*n*]. On the other hand, the strong-interaction limit (or low-density or *r*_s_ → ∞ limit) of *W*_α_[*n*] is given as^43,101,112,113^
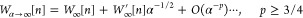
3where *W*_∞_[*n*] and *W*_∞_^′^[*n*] have a highly nonlocal density dependence, captured by the strictly correlated electrons (SCE) limit,^114−116^ and their exact evaluation in general cases is a nontrivial problem.

In particular, one of the successful attempts at practical usability of the AC DFAs came through the interaction strength interpolation (ISI) method by Seidl and co-workers^43,100,104,109,112,113,117−120^ where the DFA formula is built by interpolating between the weak- and strong-interaction regimes. The α → ∞ limit is approximated by semilocal gradient expansions (GEA) derived within the point-charge-plus-continuum (PC) model.^43,100,104,112^ Based on this form, the ISI has been tested for various applications.^106,118,121^ Also, several modifications of the ISI have been suggested^101,113,119,122,123^ as well as the PC model itself such as the hPC^124^ or modified PC (mPC),^110^ which was found to be more robust for the quasi-two-dimensional (quasi-2D) density regime.

Recently based on the ISI formula, the adiabatic connection semilocal correlation (ACSC) method was introduced,^110^ showing the alternative path of construction of semilocal correlation energy functionals. The ACSC formula interpolates the high- and low-density limits for the given semilocal DFA directly, in contrary to the standard path where the interpolation is done at the local LDA level and then corrected by gradient or meta-GGA corrections.^44,46^ We recall that in ref (110),^110^ the ACSC functional was built using the Perdew–Burke–Ernzerhof (PBE)^44^ high-density formula and mPC model showing similar or improved accuracy over its PBE precursor proving in the same time the evidence for the robustness of ACSC construction.

Motivated by the progress in this direction, this paper extends the ACSC method at the meta-GGA level and provides new insights into this context.

In the following, we briefly recall some aspects related to ACSC functional construction and investigate a few available approximations for the high- and low-density regimes. Based on that, we propose an extension of the ACSC method to the meta-GGA level using the high- and low-density limits of the Tao–Perdew–Staroverov–Scuseria (TPSS)^46^ DFA. Following that, we apply ACSC correlation energy functionals to some model systems (Hooke’s atom and H_2_ molecule) and real calculations (the atomization energies of several small molecules) to show some advantages and current limitations of ACSC functional construction. Lastly, we conclude by discussing the possible advances of the present construction.

## Theory

### Background of the Adiabatic Connection Semilocal Correlation (ACSC)

Following ref (110), the ACSC correlation energy per particle is given as (eq 15 of ref (110))
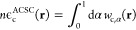
4
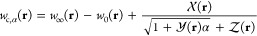
5
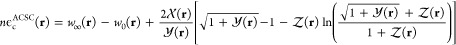
6The above expression represents a general form for the correlation energy density derived from the ISI formula^43,100,104,109,112,113,117−120^ with
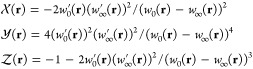
7and where *w*_0_, *w*_0_^′^, and *w*_∞_, *w*_∞_^′^ denote the approximation for energy densities for high- (or weak-interaction) and low-density (or strong-interaction) limits, respectively. Considering the accuracy of eq 4, it depends on three main aspects:

(i) The interpolation formula is used to define the *w*_c,α_(**r**) integrand in eq 4. In ref (110) (and here eq 5), the ISI interpolation formula was utilized to define ACSC. We note, however, that for this choice, the *W*_α_[*n*] contains a spurious term proportional to α^–1^ in its strong-interaction limit (α → ∞),^112^ which has been corrected in refs (101) and (115). In order, to be consistent with our previous work, we sticked with the ISI formula. Nonetheless, other possibilities also exist.^101,113,119,122^
(ii) The approximation for the α → ∞ limit. Several possibilities exist, e.g., exact treatment by employing SCE formulas (numerically expensive but feasible) or much less time-consuming variants such as mPC,^110^ hPC,^124^ or the ones derived from semilocal DFA via the procedure described in ref (112) Note that by choosing different α → ∞ limits, one can incorporate in the ACSC formula different physics, e.g., good performance for the quasi-2D regime.
(iii) The approximation for the α → 0 limit. In principle, this limit can be taken into account exactly by considering the exact exchange (EXX) and GL2 limit.^19,108,125^ However, evaluation of the GL2 correlation energy density on the numerical grid would likely be computationally quite expensive. Hence, in ref (110), the nonlocal contributions have been substituted by semilocal high-density counterparts obtained from the PBE functional.^44^

In this work, we extend the ACSC DFA by considering all input quantities at the semilocal (SL), meta-GGA level. For instance, the *w*_0_(**r**) and *w*_0_^′^(**r**) approximations are constructed as
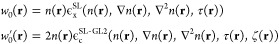
8using SL form of the GL2 correlation energy density (SL-GL2),^110^ where τ(**r**) = ∑_*j*=1_^occ^|∇ϕ_*j*_(**r**)|^2^/2 is the KS noninteracting kinetic energy density, with ϕ_*j*_(**r**) being the one-particle *j*th occupied KS orbital. We underline that the Laplacian of the density (∇^2^*n*) contains information that is is already encapsulated in τ,^126^ such that many meta-GGA XC functionals do not consider ∇^2^*n* as an ingredient.

There are also two prime motivations behind the extension of ACSC functionals to the meta-GGA level:

(i) Many of the SL-GL2 correlation energy functionals, such as TPSS-GL2 (and all TPSS-like GL2 functionals) have already been derived;^59,127^ thus, they can be easily applied in the present construction. The quantitative comparison of the accuracy of these SL-GL2 models with reference second-order GL2 correlation energy data is reported in ref (128) in Table S12.
(ii) The meta-GGA SL-GL2, such as TPSS-GL2 DFA, is one-electron self-interaction free, giving exactly zero for the hydrogen atom, which is not the case for PBE-GL2.

In the next section, we address the choice of *w*_∞_(**r**) and *w*_∞_^′^(**r**).

### TPSS-ACSC Correlation Functionals Formula

To construct ACSC meta-GGA DFA, we fix the *w*_0_(**r**) and *w*_0_^′^(**r**) (where the energy density *w*_α_(**r**) is defined by *W*_α_ = ∫d**r** *w*_α_(**r**)) in the form of TPSS exchange (*w*_0_(**r**) = *n*(**r**)ϵ_x_^TPSS^) and TPSS-GL2^59^ (*w*_0_^′^(**r**) = 2*n*(**r**)ϵ_c_^TPSS-GL2^), respectively. In the case of *w*_∞_(**r**) and *w*_∞_^′^(**r**), the choice is not so simple due to various variants available in the literature. As was noted before, the form of *w*_∞_(**r**) and *w*_∞_^′^(**r**) implies the incorporation of important physics in the ACSC formula, i.e., the quasi-2D regime via the mPC^110^ model or very accurate performance for weak and strong-interaction regime via the hPC model developed recently.^124^ However, both mPC and hPC are simple GGA-level approximations of SCE formulas, which are not one-electron self-interaction free.^124^ Therefore, the utilization of these GGA models might impact the performance of ACSC meta-GGA DFA. To overcome this limitation, one can develop the meta-GGA model for the TPSS strong-interaction^129^ regime as was done in appendix D in ref (112). Thus, for clarity of this paper, we recall that for any approximate XC energy DFA (*E*_xc_^DFA^ = *E*_x_^DFA^ + *E*_c_^DFA^), the corresponding coupling-constant integrand *W*_α_^DFA^ can be derived from the following formula
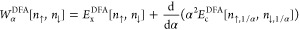
9by considering the strictly correlated α → ∞ limit.

Thus, for the low-density limit of the TPSS functional, we obtain the *W*_∞_^TPSS^ (eq 17) and *W*_∞_^′TPSS^ (eq 18) expressions with their corresponding energy densities *w*_∞_(**r**) and *w*_∞_^′^(**r**), respectively. The latter quantities incorporate all physically meaningful features, i.e., canceling one-electron self-interaction and proper behavior for the quasi-2D regime (shown later), which was also the case for the mPC model.^110^ Based on the above consideration, we construct the TPSS-ACSC correlation functional using eq 5 with TPSS variants of *w*_0_, *w*_0_^′^ and *w*_∞_, *w*_∞_^′^ energy densities.

The final TPSS-ACSC formula diverges to −∞ when *s* → 0 (*w*_0_^′^ → −∞), (e.g., for the case of the uniform electron gas (UEG) model) behaving in this limit as^110^
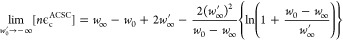
10which reveals the ACSC DFA accuracy for UEG (see also Figure 3 in ref (110) for the PBE-ACSC functional). On the other hand for *w*_0_^′^ → 0, it gives
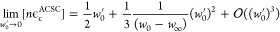
11such that *E*_c_^TPSS-ACSC^ = 0 whenever *E*_c_^TPSS-GL2^ = 0. The TPSS-GL2 correlation energy density vanish whenever τ = τ^W^, and ζ = 1, where τ^W^ is the von Weizsäcker kinetic energy density.^130,131^ Thus, for one-electron systems, where *E*_c_^TPSS-GL2^ = 0, the TPSS-ACSC correlation energy is exact, showing that the functional is one-electron self-correlation free.

**Figure 1:**
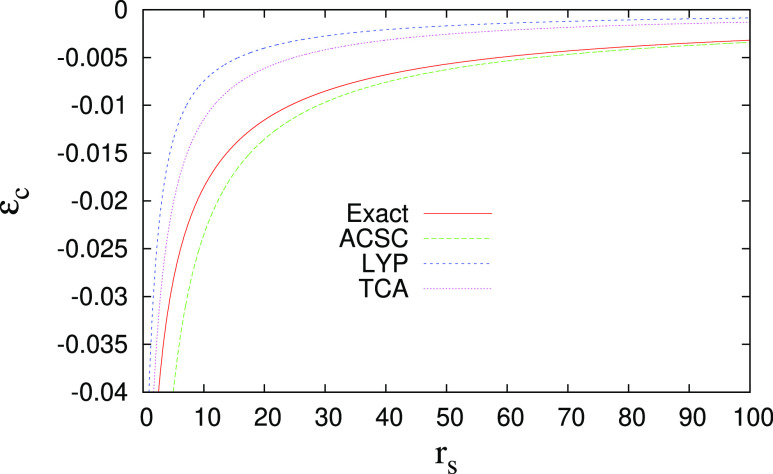
Correlation energy per particle ϵ_c_ versus the bulk parameter , for the uniform electron gas. See the text for details of the methods and exact reference curve.

At this point, analysis of the behavior of the TPSS-ACSC correlation energy is required. In Figure 1, we show the UEG correlation energies per particle of the exact LDA^132^ (shown by the exact line in Figure 1). We recall that for UEG, the reduced gradient *s* = 0; thus, *w*_0_ reduces to LDA exchange energy density and *w*_0_^′^ → −∞; thus, for the ACSC functional, we utilize the ACSC limit for UEG given by eq 10. For comparison, we also show Lee–Yang–Parr (LYP)^133^ and Tognetti–Cortona–Adamo (TCA) correlation^134−136^ energy densities. One can note that the ACSC formula is accurate in the low-density limit (*r*_s_ ≥ 20), while in the high-density limit (*r*_s_ → 0), it diverges as ∼*r*_s_^–1/2^, thus faster than the exact behavior (∼ln(*r*_s_)). Nevertheless, in the high-density limit, the exchange energy dominates over the correlation, such that this failure of the ACSC correlation should be compensated by the proper choice of the exchange functional part. This can be considered a drawback of ACSC construction because it might lead to some issues with a lack of compatibility between standard semilocal exchange functionals and the ACSC correlation functionals (mutual error cancellation effect). We will address this issue in the following.

## Results and Discussion

We first test the accuracy of *W*_∞_^TPSS^ = ∫d**r** *w*_∞_^TPSS^(**r**) and *W*_∞_^TPSS^ = ∫d**r** *w*_∞_^TPSS^(**r**) expressions, which are reported in Table 1 for real atoms. For comparison, we also present the data obtained for the exact SCE method,^17,114,115^ PC, mPC, hPC, and PBE (*W*_∞_^PBE^, *W*_∞_^′PBE^) formulas from ref (112). In the case of *W*_∞_, the TPSS approximation gives the best performance measured with respect to (wrt) SCE values (even for Ar, Kr, and Xr data reported recently^17^) being almost 3 times better than the one obtained for a very accurate hPC model. This is partially because the former correctly removes the one-electron self-interaction in *W*_∞_^TPSS^, which is taken into account in all GGA *W*_∞_ approximations. Nonetheless, even without hydrogen atom contribution (reported in parentheses), the mean absolute relative error (MARE) of TPSS *W*_∞_ presents the best performance for this model (MARE = 0.44%), closely followed by hPC (MARE = 0.48%) that are twice better than the original PC variant.

**Table 1:** Values of *W*_∞_ and *W*_∞_^′^ for Several Atoms Obtained from Different Models and Using EXX Densities^a^

	SCE	PC	hPC	mPC	PBE	TPSS
*W*_∞_						
H	–0.3125	–0.3128	–0.3293	–0.4000	–0.4169	**–0.3125**
He	–1.500	–1.463	**–1.492**	–1.671	–1.6888	–1.5122
Be	–4.021	–3.943	–3.976	–4.380	–4.4203	**–3.9803**
Ne	–20.035	**–20.018**	–20.079	–21.022	–21.2983	–19.9792
Ar	–51.555	**–51.5473**	–51.6158	–53.2709	–53.9322	–51.3799
Kr	–166.850	–167.3561	–167.4387	–170.3279	–172.0157	**–166.7765**
Xe	–322.835	–324.5206	–324.6190	–328.6846	–331.3261	**–323.3446**
MARE (%)		0.78 (0.89)	1.18 (0.48)	8.64 (5.41)	10.36 (6.53)	**0.38 (0.44)**
*W*_∞_^′^						
H	0	0.0426	0.0255	0.2918	0.243	**0**
He	0.621	0.729	**0.646**	1.728	1.517	0.728
Be	2.59	2.919	**2.600**	6.167	5.442	2.713
Ne	22	24.425	**23.045**	38.644	35.307	23.835
MARE (%)		13.71	**3.05**	130.67	104.94	10.10

a We used atomic units. The results that agree best with SCE values^114,115^ are highlighted in bold (for Ar, Kr, and Xe, the SCE *W*_∞_ values are taken from ref (17)). The last line of each panel reports the mean absolute relative error (MARE) [for *W*_∞_ (in parentheses) and *W*_∞_^′^ we report the results where H results are excluded]. The *W*_∞_^′SCE^ reference data are reported with the same precision as in ref (115).

In the case of *W*_∞_^′^ the overall performance of the TPSS model is worse than the one observed for hPC, being in line with the results reported for PC. This can be due to the fact that *W*_∞_^′TPSS^ in the slowly varying density limit does not recover correctly the gradient expansion of the PC model. The problem lies in the *H*_2_ function (eq 18), which when *t* → 0 gives rise to the term proportional to *t*^6^, in comparison to the PC model, which yields here the term proportional *t*^2^. One important difference, however, can be noted for the TPSS formula that for the H atom, it correctly recovers the SCE value, which is not possible by any GGA variant.

**Figure 2:**
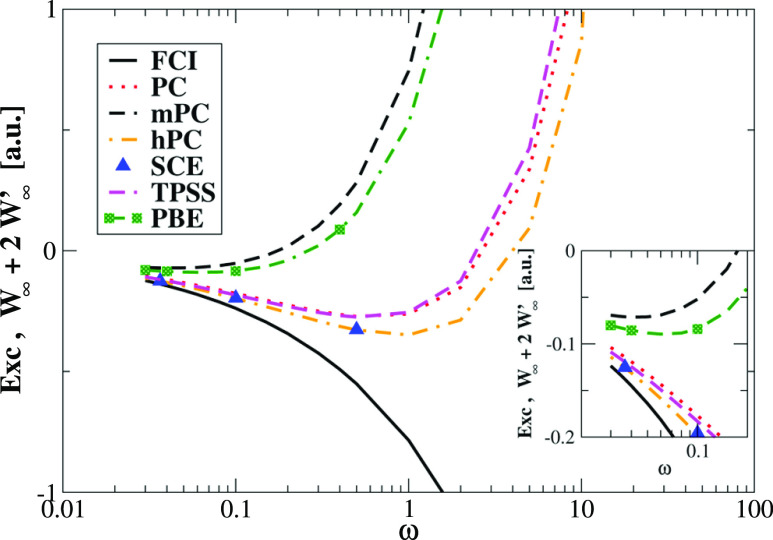
Comparison of the leading term of the XC energy (*E*_xc_ = *W*_∞_ + 2*W*_∞_^′^) in the strong-interaction regime of Hooke’s atom calculated using different models with FCI data.^128^

An additional assessment of all models is provided in Table 2 and Figure 2, where we present results obtained for Hooke’s atom at different confinement strengths ω (see further text for computational details). Turning first our attention to Table 2, we see similar trends to those presented in Table 1 for all values of ω where exact SCE data are available. Moreover, Figure 2 shows that in the small ω range (strong-interaction limit of the Hooke’s atom) hPC and TPSS yield the best estimation of the XC energy *E*_xc_ = *W*_∞_ + 2*W*_∞_^′^, being slightly better than those obtained from the PC model, while the mPC and PBE methods fail completely. Actually, mPC and PBE *W*_∞_ and *W*_∞_^′^ perform very similarly in all investigated cases, giving rise to large errors.

**Table 2:** *W*_∞_ and *W*_∞_^′^ Energies (in Ha) for Three Values of ω for Which Hooke’s Atom Has Analytical Solutions^138^ and Exact SCE Reference Data Are Available^108^^,^^a^

	SCE	PC	hPC	mPC	PBE	TPSS
*W*_∞_						
0.0365373	–0.170	–0.156	–0.167	–0.191	–0.191	**–0.170**
0.1	–0.304	–0.284	**–0.303**	–0.344	–0.344	–0.308
0.5	–0.743	–0.702	**–0.743**	–0.841	–0.843	–0.754
MARE (%)		6.78	**0.70**	12.90	12.98	0.96
*W*_∞_^′^						
0.0365373	0.022	**0.021**	**0.021**	0.060	0.053	0.026
0.1	0.054	**0.054**	0.053	0.146	0.130	0.062
0.5	0.208	0.215	**0.208**	0.562	0.501	0.240
MARE (%)		2.64	**2.13**	171.10	139.81	14.70

a The last line of each panel reports the mean absolute relative error (MARE). The bold numbers indicate the most accurate values corresponding to the reference data.

**Figure 3:**
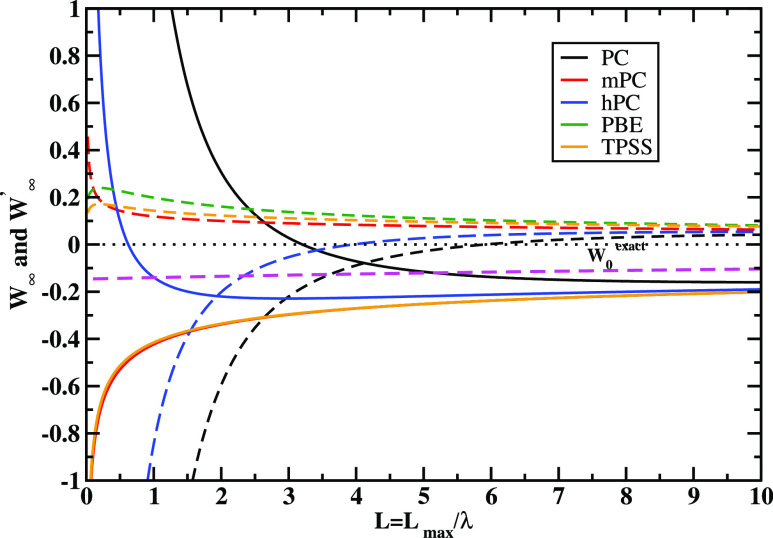
Comparison of *W*_∞_ (solid line) and *W*_∞_^′^ (dashed line) behaviors for an IBM quasi-2D electron gas of fixed 2D electron density (*r*_s_^2D^ = 4) as a function of the quantum-well thickness *L*. Also shown is the exact exchange *W*_0_. PC and mPC are obtained from PBE, and results are taken from ref (110). For PBE, the *W*_∞_ and *W*_∞_^′^ expressions are from ref (112). For TPSS, *W*_∞_ and *W*_∞_^′^ expressions are given in eqs 17 and 18, respectively.

Further, we perform the comparison of *W*_∞_ and *W*_∞_^′^ behaviors for all studied models for an infinite barrier model (IBM) quasi-2D electron gas of fixed 2D electron density (*r*_s_^2D^ = 4) as a function of the quantum-well thickness *L* as was also done in ref (110). The quasi-2D is very useful for the XC functional development, being the exact constraints in several modern density functional approximations.^6,137^ Under a uniform density limit to the quasi-2D limit, density behaves as *n*_λ_^*z*^(*x*, *y*, *z*) = λ*n*(*x*, *y*, λ*z*) and the system approaches the 2D limit when λ → ∞. In this limit, the XC energy is finite and negative, i.e., lim_λ→∞_*E*_xc_[*n*_λ_^*z*^(*x*, *y*, *z*)] > −∞. We report this in Figure 3. One can note that PC and hPC models change signs even for a mild quasi-2D regime. This feature is not allowable because it can lead to nonphysical positive correlation energy or total failure of ISI or ACSC correlation energy expressions in quasi-2D regimes. On the other hand, the mPC, PBE, and TPSS *W*_∞_ and *W*_∞_^′^ give correct behavior for a whole range of quantum-well thickness *L*.

A brief summary of all important features of strong-interaction models is given in Table 3. One can note that TPSS *W*_∞_ and *W*_∞_^′^ reproduce reference SCE data with quite a good accuracy and also some other important features, e.g., good performance in the quasi-2D regime, removing one-electron self-interaction. This possibly indicates that the description of all nonlocal features of the SCE model can be done only by the utilization of nonlocal ingredients such as τ. This is the first important finding of the present study.

**Table 3:** Brief Summarization of Properties of *w*_∞_(**r**) and *w*_∞_^′^(**r**) from Various Semilocal Models

	PC^43,112^	mPC^110^	hPC^124^	PBE^112^	TPSS^129^
level of theory	GEA	GGA	GGA	GGA	meta-GGA
accurate for the strictly correlated regime	√	×	√	×	√
quasi-2D regime	×	√	×	√	√
self-consistent calculations	×	√	√	√	√
one-electron self-interaction free	×	×	×	×	√

Now, let us turn our attention to the numerical performance of the TPSS-ACSC functional itself. In Table 4, we report the correlation energies for small atoms and molecules obtained with TPSS-ACSC and TPSS functional energy expression. In the case of atoms, the calculations are performed using the Hartree–Fock (HF) analytic orbitals of Clementi and Roetti.^143^ For molecules, we have performed the HF calculations in the ACESII^144^ program using the uncontracted cc-pVTZ^142^ basis sets and geometries taken from refs (40) and (41).We recall that the utilization of self-interaction free HF orbitals allows us to test the error specifically related to the functional construction itself, namely, the functional-driven error.^145^ As was shown in ref (146) the utilization of HF densities can sometimes lead to the worsening of predictions of DFAs or improving them for the wrong reasons. This could happen in the cases where density-driven error gives a significant contribution not canceled totally by applying the HF densities. In these cases, utilization of more accurate, correlated densities is required.^146^ However, in most semilocal DFAs, the total error is predominated by functional-driven error, meaning that HF densities are sufficiently accurate to perform such analysis.

**Table 4:** TPSS and TPSS-ACSC Correlation Energies (mHa) Divided by the Number of Electrons (*N*_e_) for 10 Atoms (Computed Using Hartree–Fock (HF) Analytic Orbitals and Densities^139−141^) and Eight Molecules (Computed Using Hartree–Fock Orbitals and Densities Obtained with Uncontracted cc-pVTZ^142^ Basis Sets)^c^

atoms	*N*_e_	TPSS	TPSS-ACSC	refs (139−141)
H	1	**0.0**	**0.0**	0
He	2	**–21.5**	–20.2	–21
Li	3	–16.5	**–15.9**	–15.1
Be	4	**–21.7**	–20.8	–23.6
N	7	**–26.5**	–25.9	–26.9
Ne	10	**–35.4**	–35.3	–39.1
Ar	18	–39.5	**–39.8**	–40.1
Kr	36	–49.2	**–49.9**	–57.4
Zn	30	–47.0	**–47.8**	–56.2
Xe	54	–54.1	**–55.3**	–57.2
MAE_atm_		2.6	**2.5**	

molecules^a^	*N*_e_	TPSS	TPSS-ACSC	ref^b^
H_2_	2	–21.1	**–19.9**	–19.8
LiH	4	–21.5	**–20.2**	–18.5
Li_2_	6	–21.0	**–20.0**	–17.9
H_2_O	10	–33.2	**–33.1**	–32.9
NH_3_	10	**–31.9**	–32.1	–30.6
HF	10	–34.3	**–34.1**	–33.7
CO	14	**–32.4**	**–32.4**	–34.0
N_2_	14	–32.7	**–32.1**	–30.6
MAE_mol_		1.7	**1.2**	

a The geometries have been taken from refs (40) and (41).

b Correlation energies obtained at CCSD(T) with the uncontracted cc-pVTZ level of theory.

c The bold numbers indicate the most accurate values corresponding to the reference data.

**Figure 4:**
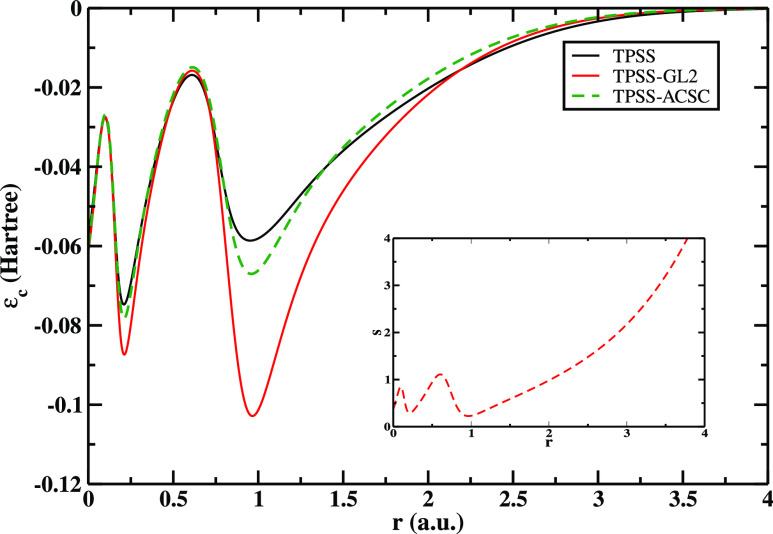
Correlation energy per particle ϵ_c_ versus the radial distance from the nucleus *r*, for the Ar atom (computed using Hartree–Fock analytic orbitals and densities^139−141^). In the inset, we show the reduced gradient .

As noted before, both considered correlation functionals are one-electron self-interaction free, which is visible in the case of the H atom. In most cases, TPSS-ACSC performs in line with its TPSS counterpart, indicating that the correlation effects are well represented in the ACSC energy expression. To visualize the correlation densities, in Figure 4, we show a comparison between ϵ_c_^TPSS^, ϵ_c_^TPSS-GL2^, and ϵ_c_^TPSS-ACSC^ for the Ar atom. Whenever *s* is small, ϵ_c_^TPSS-GL2^ starts to depart from ϵ_c_^TPSS^, diverging when *s* = 0. However, ϵ_c_^TPSS-ACSC^ is well-behaved everywhere.

As to the molecules, we note that TPSS-ACSC performs very well for the majority of systems, being slightly better than the TPSS functional. This again confirms the robustness of the correlation functional construction.

**Figure 5:**
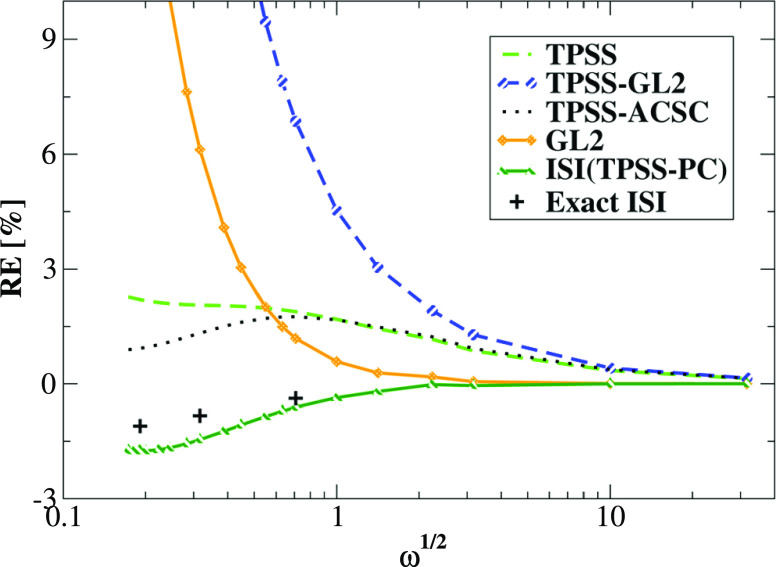
Relative error on XC energies of harmonium atoms for various values of ω computed at @EXX orbitals for several functionals using the computational setup from ref (128). The errors have been computed with respect to FCI data obtained in the same basis set.^128,148^ For all TPSS-like results, the results have been obtained together with the TPSS exchange energy functional. The GL2 and ISI(TPSS) XC correlation results are obtained with the exact GL2^18^ formula combined with EXX energy expression. The ISI formula utilizes *W*_∞_ and *W*_∞_^′^ given by eqs 17 and 18. Exact ISI data are taken from ref (108).

In Figure 5, we report the relative error (RE) on XC energy computed for the two-electron Hooke’s atom model for various values of confinement strength ω (ω ∈ [0.03, 1000]). The errors are computed with respect to full configuration interaction (FCI) results from ref (128). The calculations have been performed using an identical computational setup as in our previous study^49,128,147^ using EXX reference orbitals. We recall that for small values of confinement strength ω, the system is strongly correlated, whereas for large values of ω, we enter a weak-interaction regime. Thus, the model provides an excellent tool for testing the functional performance in these two regimes. We underline that in all following calculations, all TPSS-like correlation functionals have been combined with the TPSS exchange energy functional in order to obtain XC energies.

For medium and large values of ω, the TPSS and TPSS-ACSC functionals perform very similarly, giving in the weak interacting region a very small relative error (RE) similar to those of exact GL2 and ISI XC functionals. In a strong-interaction regime, in turn, the TPSS-ACSC improves over its TPSS precursor. We note that in the latter regime, the TPSS-ACSC functional should recover, in principle, the ISI functional data due to the inclusion in both energy expressions the *W*_∞_ and *W*_∞_^′^ in the form given by eqs 17 and 18. Although qualitatively they behave very similarly, there is a large quantitative difference between these two curves. This is most probably related to the significant impact of the GL2 term, which enters both formulas. We recall that the ISI formula utilized the exact GL2 energy expression, whereas TPSS-ACSC approximated SL variant. Although they both diverge when ω tends to zero, the origin of that behavior is different. The exact GL2 energy diverges due to closing the highest occupied molecular orbital–lowest unoccupied molecular orbital (HOMO–LUMO) gap in this regime, whereas TPSS-GL2 due to vanishing reduced gradient, which leads to a much faster divergence. This feature of TPSS-GL2 energy expression governs the behavior of TPSS-ACSC DFA in a small ω regime. Thus, we might conclude that the quantitative difference between ISI and TPSS-ACSC DFAs comes mainly from the inaccuracy of the SL-GL2 formula used in the later expression.

**Figure 6:**
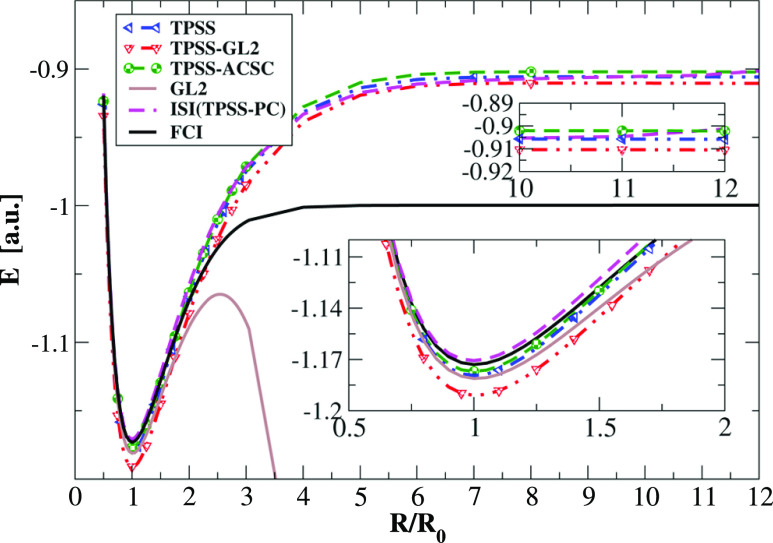
Total energy of the stretched H_2_ molecule as calculated with the various methods. The insets present the same data around the equilibrium distance (*R*/*R*_0_ = 1) and large *R*/*R*_0_ > 10 values.

Now we turn attention to another two-electron example where we may encounter a strong-interaction limit, namely, the potential energy surface for the dissociation of the H_2_ molecule, in a restricted formalism,^149^ which is one of the main DFT challenges.^149−151^ This is reported in Figure 6. All energies were obtained using EXX orbitals and densities. We want to underline that restricted HF density could give rise to substantial errors in the midbond region in the cases when the H_2_ molecule is largely stretched. As pointed out previously, the functional-driven error dominates most of the semilocal DFAs. Thus, the utilization of HF densities still gives a valid picture of the performance of semilocal DFAs for the whole range of distances of H_2_.

One can note that, in general, the TPSS-ACSC functional performs very similarly to TPSS, especially near equilibrium distance. More visible differences between these two DFAs can be seen for larger distances *R*/*R*_0_ > 3. Asymptotically, the TPSS-ACSC energy goes almost to the same value as the ISI method, with eqs 17 and 18 employed to describe *W*_∞_ and *W*_∞_^′^. This is a very interesting finding, possibly suggesting the dominant role of the strong-interaction limit (eq 10) for large separation of hydrogen atoms. We note, however, that the TPSS-GL2 total energy gives much more stable results in the asymptotic region in comparison to the exact GL2 curve, which diverges due to the closing HOMO–LUMO gap. This indicates that the proper behavior investigated here ISI and TPSS-ACSC DFAs have a different origin. In the former, the exact GL2 diverges (*E*_GL2_ → −∞), leading in the asymptotic limit to *E*_xc_^ISI^ → *W*_∞_ + 2*W*_∞_^′^(1 – 1/*q* ln(1 + *q*)) with *q* = (*E*_x_ – *W*_∞_)/*W*_∞_^′^.^118,124^ In the latter, in turn, the asymptotic limit is governed rather by the mutual error cancellation effect in the TPSS-ACSC energy expression. This is due to the fact that TPSS-GL2 energy expressions do not diverge for large *R*/*R*_0_, meaning that at the asymptotic region, eq 10 do not hold. One possible way to recover eq 10 within the TPSS-ACSC formula could be realized via proper incorporation of the local gap model^152−154^ within the SL-GL2 formula.

Let us focus on self-consistent results (@SCF) obtained within the generalized KS (gKS) scheme. As an example, we report in Table 5 AE6^155,156^ atomization energies of six small size molecules, obtained using SCF orbitals and densities. One can note that the TPSS-ACSC functional, in general, gives results that are twice worse (MAE = 18.4 kcal/mol) than for the TPSS counterpart, which yields an MAE of 7.6 kcal/mol. The same trend for the AE6 benchmark occurs when we feed the TPSS-ACSC and TPSS total energy expressions with HF orbitals. This indicates the following things:

- The major part of the error for the TPSS-ACSC functional is related to functional-driven error.^145^ This is most possibly related to the ACSC model itself, which was not designed to be accurate in the high-density limit where most of the chemical application takes place.
- Because both TPSS and TPSS-ACSC utilize the same semilocal TPSS exchange, the much larger error observed in the latter might suggest the lack of compatibility between exchange and correlation functionals (there is no error cancellation effect). The correlation energies themselves are quite accurate as shown in Table 4. This might indicate that the correct behavior of the TPSS-ACSC functional can be restored by proper design of the compatible exchange functional.

**Table 5:** AE6 Atomization Energies (in kcal/mol) Computed Using Self-Consistent (@SCF) and Hartree–Fock (@HF) Orbitals and Densities, and TPSSx Semilocal Exchange and TPSS or TPSS-ACSC Correlation Functionals^a^

	TPSS@SCF	TPSS-ACSC@SCF	TPSS-ACSC@SCF (μ = 0.40)	ref (156)
SiH_4_	334.2	337.9	332.0	323.1
SiO	187.1	189.4	179.3	191.5
S_2_	109.0	114.6	106.2	101.9
C_3_H_4_	707.8	724.0	699.8	701.0
C_2_H_2_O_2_	634.1	648.8	619.0	630.4
C_4_H_8_	1155.8	1182.8	1141.6	1143.4
MAE	7.6	18.4	6.6	

	TPSS@HF	TPSS-ACSC@HF	TPSS-ACSC@HF (μ = 0.40)	ref (156)
SiH_4_	**331.9**	337.0	332.6	323.1
SiO	179.7	**182.2**	173.1	191.5
S_2_	**103.5**	109.5	101.5	101.9
C_3_H_4_	**702.0**	719.9	698.4	701.0
C_2_H_2_O_2_	**621.1**	637.2	610.3	630.4
C_4_H_8_	**1148.3**	1179.0	1142.8	1143.4
MAE	6.2	15.3	8.5	

a The mean absolute error (MAE, in kcal/mol) is shown in the last row. The def2-QZVP basis set is used. All calculations are performed using the Q-Chem code.^157^ The bold numbers indicate the most accurate values corresponding to the reference data.

To test this possibility, we have performed ad hoc modification of the TPSS exchange functional^46^ by calibration of the second-order gradient expansion parameter (μ = 0.235). We note that in general, this parameter might vary based on the nature of the localized (such as atoms) or delocalized systems (solids). At this point, using μ = 0.40, we have observed a significant reduction of MAE for AE6 obtained at @SCF densities to 6.63 kcal/mol.

Finally, the performance of the constructed functionals is also benchmarked for other molecular test cases such as atomization energies, barrier heights and weeks, and covalent interactions. These results are reported in Table 6. A noticeable improvement is observed from TPSS-ACSC (μ = 0.40) than from TPSS-ACSC, especially for atomization energies. Interestingly, in other cases, TPSS-ACSC performs slightly better or similarly to TPSS-ACSC (μ = 0.40). This indicates that some more sophisticated modification of the TPSS exchange functional is required in order to improve the accuracy of the method for all benchmarked cases. One may note that for CT7, W17, and S22, we do not include the dispersion correction, as the inclusion of a functional specific dispersion interaction is beyond the scope of the present paper.

**Table 6:** Mean Absolute Errors (MAEs in kcal/mol) for the Benchmark Molecular Tests Obtained Using Different Methods^a^

	TPSS	TPSS-ACSC	TPSS-ACSC (μ = 0.40)
G2/148^b^	5.5	15.7	7.8
BH6^c^	8.2	8.3	8.4
HTBH38^d^	7.7	8.3	7.1
NHTBH38^d^	9.2	9.2	9.1
CT7^e^	2.0	1.7	1.1
WI7^e^	0.24	0.26	0.12
S22^f^	3.4	4.1	5.5

a All calculations are performed self-consistently using a def2-QZVP basis set with the Q-Chem code.^157^

b Atomization energies of 148 molecules.^158^

c Six barrier heights.^156^

d 38 hydrogen (HTBH38) and 38 nonhydrogen bonded reaction barrier heights (NHTBH38).^159^

e Seven charge transfer molecules, and seven weekly interacting test sets.^160^

f 22 noncovalent interacting systems.^161^

### Conclusions

In this work, we have constructed a semilocal meta-GGA correlation energy functional, based on the ACSC method proposed in ref (110). The correlation functional, denoted as TPSS-ACSC, interpolates the high- and low-density limit of the popular TPSS correlation energy functional showing some direction on how to incorporate a strong-interaction regime within the approximate, semilocal exchange–correlation formula.

The new correlation TPSS-ACSC functional is nonempirical, one-electron self-interaction free accurate for small atoms and molecules. We provide a careful assessment of the TPSS-ACSC functional base on some model systems (the uniform electron gas, Hooke’s atom, stretched H_2_ molecule) and real-life calculations (atomization energies) showing some advantages and disadvantages of ACSC construction. From this broad perspective, we can conclude that, although the ACSC method holds promise for proper description of a strong-interaction regime, it is still in its infancy, which implies that there is still much space for improvement. The most important conclusions of this study are as follows:

- The strong-interaction limit obtained from the semilocal TPSS functional formula (*W*_∞_^TPSS^ and *W*_∞_^′TPSS^) reproduces quite well reference SCE data. Moreover, both possess some other important features, e.g., good performance in the quasi-2D regime and removing one-electron self-interaction. Thus, both formulas could be effectively applied in the construction of ACSC and ISI-like formulas.
- Although our numerical tests suggest that the strong-interaction limit of semilocal TPSS-ACSC correlation is well represented, the semilocal GL2 part may need some amendment (Hooke’s atom, stretched H_2_ molecule cases), e.g., via proper incorporation of the local gap model.^152−154^
- In order to improve the accuracy of the TPSS-ACSC XC functional, it must be combined with the compatible exchange functional leading to a much better balance in the XC term (better mutual error cancelation effect). As was shown the ad hoc modification of TPSS exchange gives some hints in that direction.

Some of these new developments in the ACSC context will be addressed in a future study.

## Data Availability

The data that support the findings are published within this study.

## Details of the TPSS-ACSC Correlation Functional

Here, we summarized the expressions of energy density *w*_α_ of eqs 4–7, which is defined by *W*_α_ = ∫d^3^*r w*_α_(**r**). Thus, one required following energy densities *w*_0_(**r**), *w*_0_^′^(**r**), *w*_∞_(**r**), and *w*_∞_^′^(**r**) to calculate the ACSC correlation.

We take in the following expressions:

(i) First, *w*_0_(**r**) = *n*(**r**) ϵ_*x*_^TPSS^(**r**), where ϵ_*x*_^TPSS^(**r**) is the TPSS exchange energy per particle^46^ given by
  12
  with κ = 0.804. See ref (46) for the details of the TPSS exchange enhancement factor (*F*_*x*_^TPSS^).
(ii) Second, *w*_0_^′^(**r**) = 2*n*(**r**)ϵ_*c*_^TPSS-GL2^, where ϵ_c_^TPSS-GL2^ is the Görling–Levy second-order limit of the TPSS correlation energy^46^ per electron, which is given by^59^
  13
  where *d* = 2.8 hartree^–1^ is a constant and
  14
  In eq 14, ϵ_c_^PBE-GL2^ is the Görling–Levy limit of the PBE correlation energy per electron. It is obtained by replacing λ**r** with **r** in the λ → ∞ uniform density scaling limit of the PBE correlation energy per electron and has the expression
  15
  where γ = (1 – ln 2)/π^2^, ϕ(ζ) = 1/2[(1 + ζ)^2/3^ + (1 – ζ)^2/3^], *s* = |∇*n*|/2*nk*_F_ is the reduced density gradient, *k*_F_ = (3π^2^*n*)^1/3^, and χ = (β/γ)*c*^2^*e*^–^ω/γ ≈ 0.72161, where *c* = (3π^2^/16)^1/3^, β = 0.066725, and ω = 0.046644.
  The spin-dependent function ϵ̃_c,σ_^PBE-GL2^ is defined as
  16
  The function *C*(ζ, ξ) is the spin-dependent function, where ζ is the spin-polarization and ξ = |∇ζ|/2*k*_F_.
(iii) Third, in the case of TPSS XC functional, the *W*_∞_ is derived as
  17
(iv) Fourth and finally, *W*_∞_^′^ for the TPSS functional reads
  18
  where *d*_1_(ζ) = 1.5 (spin-independent) was fixed using the same reasoning as in ref (112) and *E*_x_^TPSS^[*n*_↑_, *n*_↓_] is the spin-resolved TPSS exchange.^46^*H*_1_ and *H*_2_ are the same as given by eqs D11 and D12 of ref (112)*C*(ζ, ξ) is given in eq 14 of ref (46). We recall that *W*_∞_^TPSS^[*n*_↑_, *n*_↓_] was already reported in ref (129). However, the expression of *W*_∞_^′TPSS^[*n*_↑_, *n*_↓_] can be obtained in a similar fashion to eq D16 of PKZB expression.^112^ For the details of the parameters and terms, see ref (112) (for *d*_0_(ζ), *d*_1_(ζ), and *d*_1_(1)) and ref (46) (for *C*(ζ, ξ)). One may note that the expressions of TPSS (given in this paper) differ from PKZB (given in ref (112)) from their correlation point of view.
